# Development of a nanobody-based immunoassay for the sensitive detection of fibrinogen-like protein 1

**DOI:** 10.1038/s41401-020-00574-4

**Published:** 2021-02-25

**Authors:** Wan-ting Zhang, Ting-ting Liu, Man Wu, Xiao-chen Chen, Li Han, Zhen-zhong Shi, Yu-ying Li, Xi-yang Li, Hai-xing Xu, Li-kun Gong, Pei-hu Xu, Yong Geng

**Affiliations:** 1 School of Chemistry, Chemical Engineering and Life Sciences, Wuhan University of Technology, Wuhan, 420070 China; 2 Shanghai Institute of Materia Medica, Chinese Academy of Sciences, Shanghai, 201203 China; 3 School of Pharmacy, Shanghai University of Traditional Chinese Medicine, Shanghai, 201203 China; 4 College of Food Science and Technology, Shanghai Ocean University, Shanghai, 201306 China; 5 University of Chinese Academy of Sciences, Beijing, 100049 China; 6 School of Novel Chinese Medicine, Nanjing University of Chinese Medicine, Nanjing, 210046 China; 7 Zhongshan Institute for Drug Discovery, Institutes of Drug Discovery and Development, Chinese Academy of Sciences, Zhongshan, 528400 China

**Keywords:** nanobody, FGL1, cancer immunology, diagnosis, immune checkpoint

## Abstract

Immune checkpoint inhibition is an important strategy in cancer therapy. Blockade of CTLA-4 and PD-1/PD-L1 is well developed in clinical practice. In the last few years, LAG-3 has received much interest as an emerging novel target in immunotherapy. It was recently reported that FGL1 is a major ligand of LAG-3, which is normally secreted by the liver but is upregulated in several human cancers. FGL1 is a crucial biomarker and target for cancer immunotherapy. As the efficacy of immunotherapy is limited to specific types of patients, the subset of patients needs to be selected appropriately to receive precise treatment according to different biomarkers. To date, there is no test to accurately assess FGL1 expression levels. Nanobodies have some outstanding features, such as high stability, solubility and affinity for diagnostic and therapeutic applications. Here, we report the development and validation of a rapid, sensitive, and cost-effective nanobody-based immunoassay for the detection of FGL1 in human serum. In this study, human FGL1 recombinant protein was expressed and purified for the first time as an immunized antigen. Then, we constructed a nanobody phage display library and screened several nanobodies that bind FGL1 with high affinity. We selected two nanobodies targeting different epitopes of FGL1, one as a capture and the other conjugated with HRP as a probe. The double nanobody-based sandwich ELISA to detect the concentration of FGL1 showed a good response relationship in the range of 15.625–2000 ng/mL, and the recoveries from the spiked sample were in the range of 78% and 100%. This assay could be used as a potential approach for evaluating FGL1 expression for patient stratification and for predicting the therapeutic efficacy of targeting the LAG3/FGL1 axis.

## Introduction

In the last few years, immune checkpoint blockade has been considered a revolutionary therapeutic strategy in cancer immunotherapy [1, 2]. Many checkpoint inhibition therapies are under rapid development in preclinical or clinical stages [3–5]. Cancer immunotherapies targeting the PD1/PDL-1 axis have been approved by the FDA for the treatment of multiple types of tumors; they induce clinically durable responses and consequently extend overall progression-free survival [1, 3]. However, currently, significant responses to immunotherapies are limited to a minority of patients, and some patients develop treatment resistance or experience high toxicity. Therefore, there exists a clinical need for the identification of more immune checkpoint pathways [1].

Lymphocyte-activation gene 3 (LAG-3) is mainly expressed on activated T cells as an inhibitory receptor and is significantly upregulated, together with other inhibitory receptors such as PD-1, TIGIT, TIM3, and CD160, in many types of cancer, which induces the exhaustion of T cells in malignant tumors [6–9]. In this regard, LAG-3 has attracted widespread attention as a new target. The modest effect of single LAG-3 blockade was evident in clinical trials, while dual blockade with LAG3/PD-1 compounds demonstrated a promising, durable response and overall survival with acceptable toxicity [10, 11].

However, it is controversial whether MHC-II is the major ligand mediating the immunosuppressive function of LAG-3, and recently, fibrinogen-like protein 1 (FGL1) has been reported as its major inhibitory ligand, which is normally secreted by the liver [12]. The study showed that the FGL1-LAG-3 interaction displayed a high *K*_D_ value of 1.5 nM. FGL1 also inhibited the antigen-specific T cell response via LAG-3 in vitro and in vivo, and FGL1-KO enhanced T cell immunity and suppressed tumor growth in a mouse model [12]. Moreover, tumor immunity was stimulated by the antibody blocking the FGL1-LAG-3 interaction in the tumor microenvironment [12]. In addition, the authors found that FGL1 was overexpressed in some human cancers and that a high concentration of FGL1 in plasma was associated with poor prognosis and therapeutic outcomes in patients treated with anti-PD therapy; moreover, the anti-FGL1 antibody showed synergistic efficacy with anti-PD therapy [12]. Finally, the authors proposed that the FGL1-LAG-3 pathway was an important immune escape mechanism and a potential target for checkpoint inhibitor-based cancer immunotherapy [12].

The success of immunotherapy first requires a thorough study of the tumor microenvironment to identify individual candidate patients for optimization of anticancer therapy and precision medicine [13–15]. Trials in which therapies were tailored based on biomarker profiling indicated that matched therapy achieved a higher objective response rate and a lower toxicity [13, 16, 17]. From a clinical perspective, patients’ circulating FGL1 may provide information on the activation status of the FGL1-LAG-3 pathway and serve as a diagnostic and prognostic biomarker in clinical studies to design and detect innovative immunotherapies [18–20]. In addition, the analysis of FGL1 in serum as a liquid biopsy is a simple and noninvasive alternative to surgical biopsies that allows doctors to gather a range of tumor-related information and provides clues to the most effective immunotherapy. Moreover, liquid biopsy is much easier for patients to tolerate [21]. Most importantly, this approach might be more sensitive to disease progression than imaging scans at a stage before the tumor would cause clinical symptoms [21, 22]. To the best of our knowledge, however, there is currently no serum FGL1 assay suitable for clinical studies.

Nanobodies (Nbs) are recombinant minimal antigen binding fragments derived from heavy-chain-only antibodies, which are present in animals from the Camelidae family and Cartilaginous fish [23–25]. Because of their unique characteristics, such as small size, high stability, solubility, and great affinity for their ligands, they have received much attention as attractive alternatives for antibodies, especially suitable for applications in biomedical research, diagnosis, and treatment [26–29]. In addition, they can be highly expressed in prokaryotic systems and are easily engineered, enhancing their potential as low-cost immunoaffinity assay reagents [30, 31].

Here, we report the generation and characterization of nanobodies with specificity for human FGL1 and develop a double nanobody-based sandwich ELISA for the detection of FGL1 in serum. This assay is much simpler than existing methods for determining the activity status of the FGL1-LAG-3 pathway and for designing and detecting new LAG-3/FGL1-related cancer immunotherapies based on patients’ circling FGL1, which might be convenient for use in clinical trials.

## Materials and methods

### Constructs, expression, and purification of human FGL1

Full-length human FGL1 (amino acid 1-306, NCBI accession NM_004467) was subcloned into a modified pFastBac expression vector containing a C-terminal Fc tag separated by a 3 C protease cleavage site to facilitate its removal, and a Flag tag was engineered at the C-terminus of the construct for affinity purification. Human FGL1 was expressed in HEK293S GnTI- cells using the baculovirus infection system. For protein expression, HEK293S GnTI- cells grown in suspension at 37 °C in FreeStyle 293 Expression Medium (Gibco, San Diego, CA, USA) supplemented with 2% fetal bovine serum (FBS) were transduced with baculovirus (10%, *v*/*v*) and supplemented with 10 mM sodium butyrate at 30 °C when the cell density reached 2.5 × 10^6^ cells/mL. The supernatant was collected at 72 h post transduction. FGL1 protein was isolated from the cell supernatant by anti-Flag antibody (M2) affinity chromatography and eluted with 0.2 mg/mL Flag peptide in 50 mM Tris, pH 8.0 and 150 mM NaCl. The protein was further purified by gel filtration chromatography (Superdex 200, GE Healthcare Life Sciences, Piscataway, NJ, USA) in 20 mM Tris, pH 8.0 and 150 mM NaCl to remove aggregates. The FGL1-Fc fusion protein was digested with 3 C protease overnight at 4 °C, and the mixture was passed through the anti-Flag M2 column to remove the Fc and then passed through a Ni-NTA agarose column to remove the 3 C protease. FGL1 protein was obtained from the flow-through.

### Nanobody expression and purification

Nanobodies were encoded in pMECS vectors for expression in TOP10F’ *Escherichia coli* cells. Briefly, the precultures were grown in 10 mL of LB media supplemented with 100 μg/mL ampicillin, 2% (wt/vol) glucose, and 1 mM MgCl_2_ in a sterile 50-mL Falcon tube at 37 °C and 220 rpm for 7–8 h. The preculture was inoculated into 1 L 2TY media containing 100 μg/mL ampicillin, 0.1% (wt/vol) glucose, and 1 mM MgCl_2_, and then the mixture was incubated at 37 °C and 220 rpm until reaching an *OD*_600 nm_ of 0.6–0.8, followed by induction with 1 mM IPTG at 28 °C and 220 rpm for 12 h. The bacteria were harvested by centrifugation for 45 min at 5000 × *g* and resuspended in 50 mL of Buffer A (50 mM Tris-HCl, 150 mM NaCl, pH 8.0, 1 mM PMSF) and lysed by sonication. The lysate was spun down for 45 min at 10,000 × *g*, and the supernatant was loaded on a gravity column with 5 mL Ni-NTA agarose resin. The nanobody-bound resin was washed with 50 mL Buffer A containing 20 mM imidazole and then eluted with 25 mL Buffer A containing 300 mM imidazole. Finally, the eluate was fractionated by Superdex-75 gel filtration chromatography (GE Healthcare Life Sciences, Piscataway, NJ, USA) in gel filtration buffer (20 mM Tris-HCl, 150 mM NaCl, pH 8.0).

### Bactrian camel immunization and construction of the immune library

A nanobody library was generated as described previously [32]. Briefly, a healthy young bactrian camel was immunized subcutaneously with 1.5 mg of human FGL1 recombinant protein in an equal volume of Gerbu FAMA adjuvant seven times at weekly intervals. After the last immunization, peripheral blood lymphocytes (PBLs) were isolated by Ficoll-Paque Plus according to the manufacturer’s instructions, and total RNA was extracted from PBLs using an RNeasy Plus Mini Kit (Qiagen, Germantown, MD, USA) and transcribed to cDNA through reverse transcription PCR with a Super-Script^TM^ III FIRST-Strand SUPERMIX Kit (Invitrogen, Carlsbad, CA, USA). Then, the VHH fragment was amplified with two-step enriched-nested PCR using the following primers: CALL-leader primer: 5′-GTCCTGGCTGCTCTTCTACAAGG-3′, CALL-CH2 primer: 5′-GGTACGTGCTGTTGAACTGTTCC-3′, VHH-reverse primer: 5′-GATGTGCAGCTGCAGGAGTCTGGRGGAGG-3′, VHH-forward primer: 5′-CTAGTGCGGCCGCTGAGGAGACGGTGACCTGGGT-3′. The final product was digested by *Pst* I and *Bst*E II and then ligated with 10 µg pMECS vector overnight at 16 °C. The ligation product was purified with a PCR purification kit (Tiangen BIOTECH, Beijing) and electroporated into electrocompetent *E. coli* TG1 cells. The recovered transformed cells were diluted and plated on solid 2TY medium supplemented with 100 µg/mL ampicillin and 2% glucose. The size of the constructed nanobody library was evaluated by counting the number of bacterial colonies. In addition, the nanobody insertion rate was analyzed by PCR amplification. Cells were collected from plates and stored at −80 °C.

### Screening nanobodies against phage display

Nanobodies against the FGL1-Fc fusion protein were selected by the phage display strategy as previously reported [32]. Briefly, 400 µL of library stock was inoculated in 65 mL of 2TY medium, incubated at 37 °C and 200 rpm, and then superinfected with M13KO7 helper phage at a ratio of 20:1 helper phage to TG1 cells. After incubation for 30 min without shaking, the medium was changed to 2TY medium containing 100 μg/mL ampicillin and 50 μg/mL kanamycin, and then the cells were cultured overnight. The supernatant was harvested, and phages were precipitated twice with 0.25 volumes of 20% PEG6000 in 2.5 M NaCl and resuspended in ice-cold phosphate-buffered saline (PBS). Then, 100 µL of FGL1 protein (10 μg/mL) was dispensed in four nonadjacent wells of MaxiSorp 96-well immunoplates and incubated overnight at 4 °C. The wells were washed with PBST0.05 three times, and then blocking solution was added and incubated for 2 h at room temperature.

The phage was dispensed into FGL1-Fc-coated wells and incubated for 2 h on a vibrating platform (700 rpm). The wells were washed 10 times with PBST0.05 and 5 times with PBS. The bound phages were eluted with 0.25 mg/mL trypsin, collected and then used for titration and subsequent amplification in *E. coli* TG1 cells for consecutive rounds of panning. After three rounds of biopanning, we screened 60 phages with affinity for FGL1-Fc from the third-round sublibrary. Afterward, nanobodies targeting the epitope of FGL1 were selected by ELISA. Finally, the selected phages were divided into different families according to the sequence of amino acids in complementary determining region 3 (CDR3).

### Selection of detecting nanobody

We selected three nanobodies (NB11, NB16, NB23) as candidate detection nanobodies, for which the CDR3 regions were completely different from that of NB29 according to sequence alignment. It was further confirmed that they have different epitopes of FGL1 using ELISA. Three nanobodies (4 µg/mL) were added to different wells of a MaxiSorp 96-well plate and incubated overnight at 4 °C. After blocking, FGL1 protein was added to each well at different concentrations (20, 5, 1, and 0 µg/mL). Then, HRP-NB29 solution was added to the plate to form a “capture Nb-FGL1-HRP-NB29” complex. After washing, 100 μL of a mixture of TMB and hydrogen peroxide was added to each well for 15-20 min. The reaction was stopped with 2 M H_2_SO_4_. Absorbance at 450 nm was measured using a microplate reader.

### Affinity measurement

The binding affinity of FGL1 to the nanobodies was measured using a Biacore S200 device (GE Healthcare Life Sciences, Piscataway, NJ, USA). The FGL1 protein flowed through the negatively charged chip at a concentration of 1 mg/mL and a flow rate of 10 μL/min for 1 min and was captured by an amino-carboxyl coupling reaction. Subsequently, the nanobody was passed through the chip at a series of concentrations. All Biacore kinetic experimental data were obtained using Biacore S200 Evaluation Software to calculate the *K*_D_, which is the ratio of *k*_d_/*k*_a_.

### Evaluation of thermal and chemical stability

NB29 and NB16 were heated at different temperatures (4, 20, 35, 50, 65, and 95 °C) for 5 min and at 90 °C for different lengths of time (5, 15, 30, 45, 60, 75, and 90 min). Then, the samples were cooled to RT and examined for their binding capacity to FGL1 by ELISA. The chemical stability of NB29 and NB16 was tested by ELISA at different pH values (2, 3, 5, 7, 8, and 10) for 2 h.

### HRP coupling experiment

HRP (horseradish peroxidase) conjugation to NB29 (or NB16) was carried out by the following steps [33]. Ten microliters of fresh NaIO_4_ (260 mM) was premixed with 130 μL 0.2 M acetate buffer (pH 5.4). Then, 70 μL of the mixture was mixed with 60 μL of 20 mg/mL HRP, placed on ice in the dark and incubated for 30 min with shaking (100 rpm). Then, the reaction was stopped by immediate gel filtration on a Zeba spin desalting column (7 K MWCO, Pierce 89882, Rockford, lL, USA) pre-exchanged with Milli-Q water. NB29 (or NB16) was added to periodate-oxidized HRP at a molar ratio of 1:1, and a 1/20 total volume of 0.2 M sodium carbonate buffer (pH 9.5) was added to raise the pH. The coupling was performed in the dark at room temperature for 2 h with shaking (600 rpm). Then, 1/100 total volume of 5 M sodium cyanoborohydride in 1 M NaOH was added to reduce the Schiff base formed to secondary amine. After 30 min incubation, 1/10 volume (28 μL) 2 M glycine in PBS was added to block unreacted aldehyde sites. After another 30 min incubation, the sample was passed through a 0.22 μm filter, and the resultant conjugate mixture was further separated with a gel filtration chromatography column (Superdex 200, GE Healthcare Life Sciences, Piscataway, NJ, USA). To remove free HRP, FPLC peak fractions were collected, diluted, and then purified using spin column filled with Ni-NTA resin.

### Nanobody-based immunoassay for the detection of FGL1

First, NB29 was labeled with biotin using the EZ-Link Sulfo-NHS-LC-Biotinylation kit (Pierce 21935, Rockford, lL, USA). The newly prepared 10 mM Sulfo-NHS-LC-Biotin was added to the NB29 solution and incubated on ice for 2 h. The protein sample was then buffer-exchanged to remove the excess biotin reagent using a desalting column.

Then, streptavidin (2 µg/mL) was added to different wells of a MaxiSorp 96-well plate, incubated overnight at 4 °C and blocked with 3% BSA. Biotin-NB29 was added to wells and incubated for 1 h. Then, FGL1 protein was diluted to a series of different concentrations (2000, 1000, 750, 500, 350, 250, 125, 62.5, 31.25, and 15.625 ng/mL) and dispensed into different wells, while quality control samples (750, 500, and 100 ng/mL), spiked samples, and specificity test samples (CD24, siglet 15, and AXL, 100 ng/mL) were added into other wells, and the plate was incubated for 1 h. After washing, 100 µL of 50-fold diluted HRP-conjugated NB16 solution was added to each well and incubated for 1 h. After washing three times, the mixture of TMB and hydrogen peroxide was added for 15–20 min, and the reaction was stopped with 2 M H_2_SO_4_. Finally, the absorbance was read at 450 nm.

### Statistical analysis

The data were analyzed by GraphPad Prism 7 (GraphPad Software Inc., San Diego, CA, USA) and the results were presented as mean ± SEM.

## Results

### Human FGL1 expression and purification

**Fig. 1:**
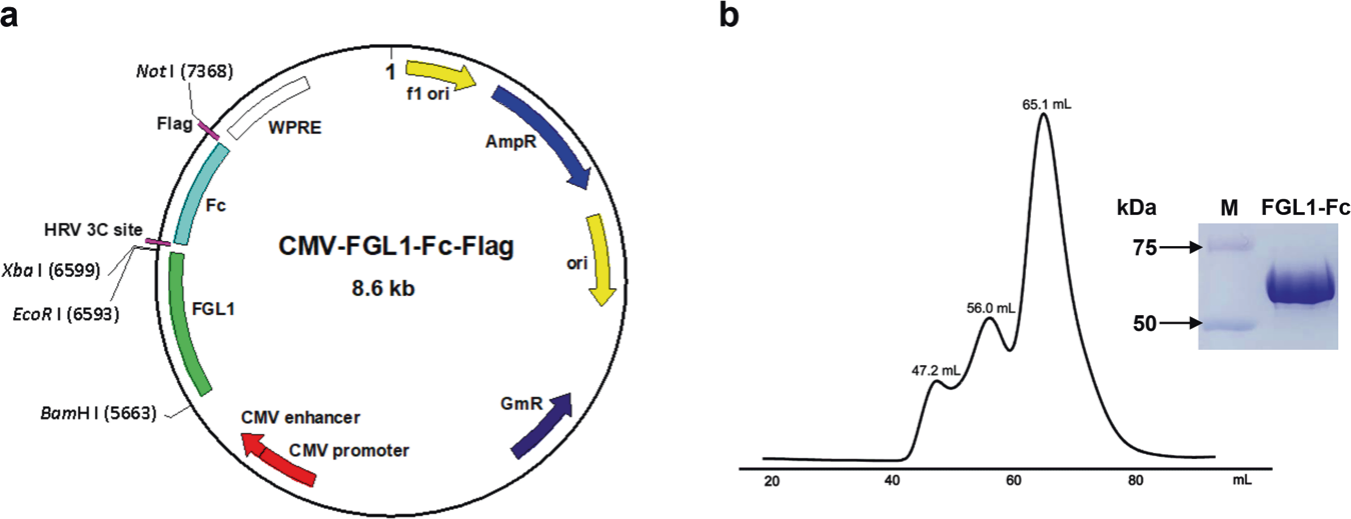
Purification of FGL1. **a** Construction map of the FGL1 expression vector. **b** Size-exclusion chromatography profile and SDS-PAGE of purified FGL1.

The human FGL1 protein contains 312 amino acids, comprising a signal peptide, a coiled-coil domain at the N-terminus and a fibrinogen domain at the C-terminus that interacts with LAG-3. FGL1 was secreted from cells as a disulfide-tethered homodimer and two pairs of disulfide bonds in each protomer. Because of the complexity of its structure, FGL1 needs to be expressed in mammalian cells to ensure proper folding and posttranslational modification. We tested the expression of FGL1 fragments of different lengths in HEK293S GnTI- cells using the baculovirus infection system, but the expression level was very low. The human IgG1 Fc fragment was inserted into the C-terminus of FGL1 to increase its expression and facilitate purification; and the sequence of 3C protease sequence and linker (GGGGS) were also inserted between the FGL1 and Fc fragments, followed by a Flag tag (Fig. 1a). Western blot data showed that the amount of FGL1 expression was significantly increased for the Fc fragment fusion protein. The secreted protein was subjected to M2 affinity chromatography and purified using size exclusion chromatography. The experiment showed a larger fraction of well-folded secreted FGL1-Fc with multiple states, including one aggregate peak and two FGL1 peaks (Fig. 1b). The peak at approximately 66 mL corresponds to the desired homodimer of FGL1, which was further verified as a band of ~60 kDa on SDS-PAGE (Fig. 1b).

The FGL1-Fc fusion protein was digested with 3 C protease and subjected to M2 affinity chromatography.

### Construction of the anti-FGL1 nanobody immune library

**Fig. 2:**
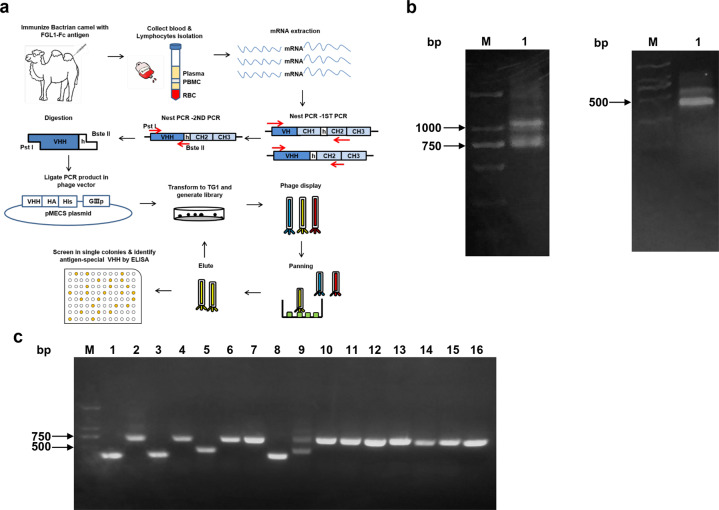
Construction of FGL1-specific immune nanobody library. **a** Schematic illustration of screening FGL1-specific nanobodies. **b** Nanobody gene fragments were amplified by a first PCR (left) and a second PCR (right). Bands of 1000 bp and 700 bp appeared after one round of PCR, and a band of ~450 bp appeared after the second round of PCR. **c** The correct insertion rate of the library was estimated by analyzing the PCR product of randomly selected colonies.

The general process of constructing the immune library and biopanning for FGL1-specific nanobodies is shown in Fig. 2a. Total RNA was extracted from PBLs of the FGL1-immunized bactrian camel and transcribed to cDNA. The VHH gene-encoding sequence was amplified through two-step nested PCR. The first PCR product consisted of two fragments, 700 bp and 1000 bp (Fig. 2b), with the former representing the nanobody encoding gene. This 700 bp fragment was separated and reamplified as the template for the second PCR, generating a 450 bp fragment corresponding to the VHH gene sequence (Fig. 2b). The VHH gene was digested with the restriction enzyme, inserted into the phage display vector and transfected into TG1 cells. The nanobody library size was calculated by counting the colonies and multiplying the number of colonies with the corresponding dilution factor. The library against FGL1 was 1.04 × 10^8^ clones (CFU/mL). To evaluate the efficiency of insertion, we analyzed the PCR products of the transformants and found that 75% of these had a nanobody insert (Fig. 2c). Our experiment showed that the FGL1-specific nanobody immune library was successfully constructed.

### Selection of nanobodies specific for human FGL1

**Fig. 3:**
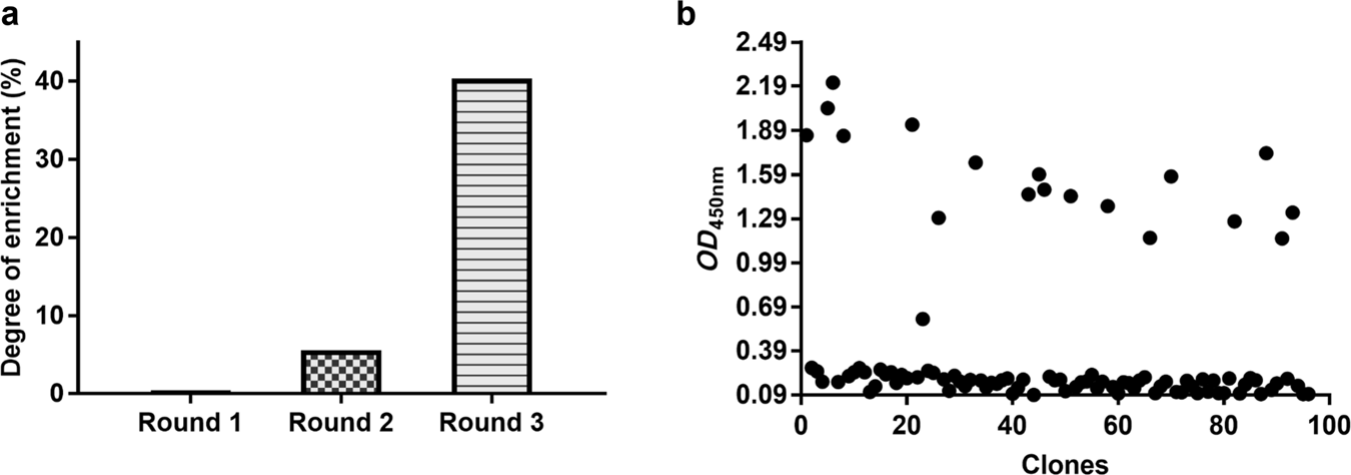
Screening of anti-FGL1 nanobodies by panning. **a** The anti-FGL1 nanobodies were enriched ~40-fold after three rounds of panning. **b** Identification of anti-FGL1-specific nanobodies by ELISA. The clones with an absorbance value more than three times that of the control group were determined as binding phages.

After the phage display library was rescued and amplified with M13KO7 helper phage, the library was subjected to consecutive rounds of panning on human FGL1-Fc-coated nonadjacent wells of a MaxiSorp 96-well plate, and the selection condition for enrichment became increasingly stringent with successive rounds of panning. After three rounds of panning, the fold enrichment of FGL1-specific VHHs reached 40 (Fig. 3a). We randomly inoculated 95 individual clones into wells of a 96-well round-bottom culture plate and incubated them overnight to prepare master plates. The periplasmic extracts from these clones were screened for specific binding to FGL1 protein by ELISA. The results revealed that the *OD*_450 nm_ value of 60 clones was twice as high as that of the blank control (Fig. 3b). These nanobodies were grouped into families according to the differences in CDR3 sequence. The highest reading, indicating the strongest binding with FGL1, was observed for NB29.

### Selection of the detection nanobody paired with NB29

**Fig. 4:**
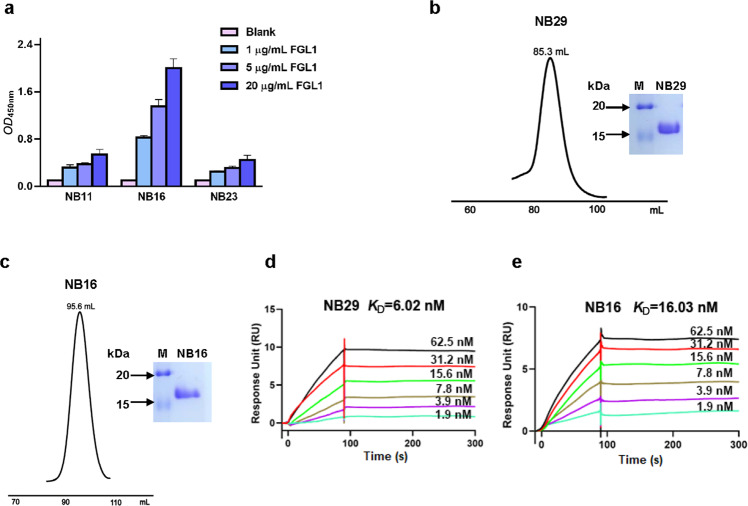
Pairwise selection, purification, and binding capacity of Nb for FGL1 detection. **a** Selection of nanobodies binding to different epitopes of FGL1. Different concentrations (0, 1, 5, and 20 µg/mL) of FGL1 were added to each well, and the same amount of HRP-NB29 was added. The absorbance value at *OD*_450 nm_ was analyzed. Size-exclusion chromatography profile and SDS-PAGE of purified NB29 (**b**) and NB16 (**c**). (**d, e**) SPR analysis of NB29 and NB16 over a range of concentrations (1.9, 3.9, 7.8, 15.6, 31.2, and 62.5 nM). The response value reflects the binding affinity between FGL1 and Nbs.

Based on the rationality of the direct nanobody-based ELISA, the detecting nanobody should have different epitopes than the detector nanobody. Three nanobodies were randomly selected and individually coated on the microplate, followed by the addition of FGL1- and HRP-conjugated NB29. As shown in Fig. 4a, the value of NB16 depended on the concentration of FGL1, indicating that NB16 and NB29 bound two different sites on FGL1. To expose the capture nanobody to FGL1, it was biotinylated by a biotinylated kit as a capturing nanobody.

### Binding kinetics measurement of FGL1 with purified Nbs

To evaluate the binding activity between the purified nanobodies and FGL1, nanobodies such as NB16 and NB29 were expressed and purified. The SEC profile demonstrated a single symmetrical peak, indicating the good solubility and homogeneity of the nanobodies, and the result of SDS-PAGE showed a single band of ~15 kDa, as expected for the size of NB29 and NB16 (Fig. 4b and c). Then, we detected the equilibrium dissociation constant (*K*_D_) between the nanobodies and FGL1 using surface plasmon resonance (SPR). NB29 displayed a high binding affinity for FGL1, with a *K*_D_ of 6.02 nmol/L, and NB16 also showed a sensitive binding affinity for FGL1, with a *K*_D_ of 16.03 nmol/L (Fig. 4d and e). Our results indicated that NB16 could be used as an ideal sensor and that NB29 could be used for FGL1 capture.

### Analysis of thermal and chemical stability

**Fig. 5:**
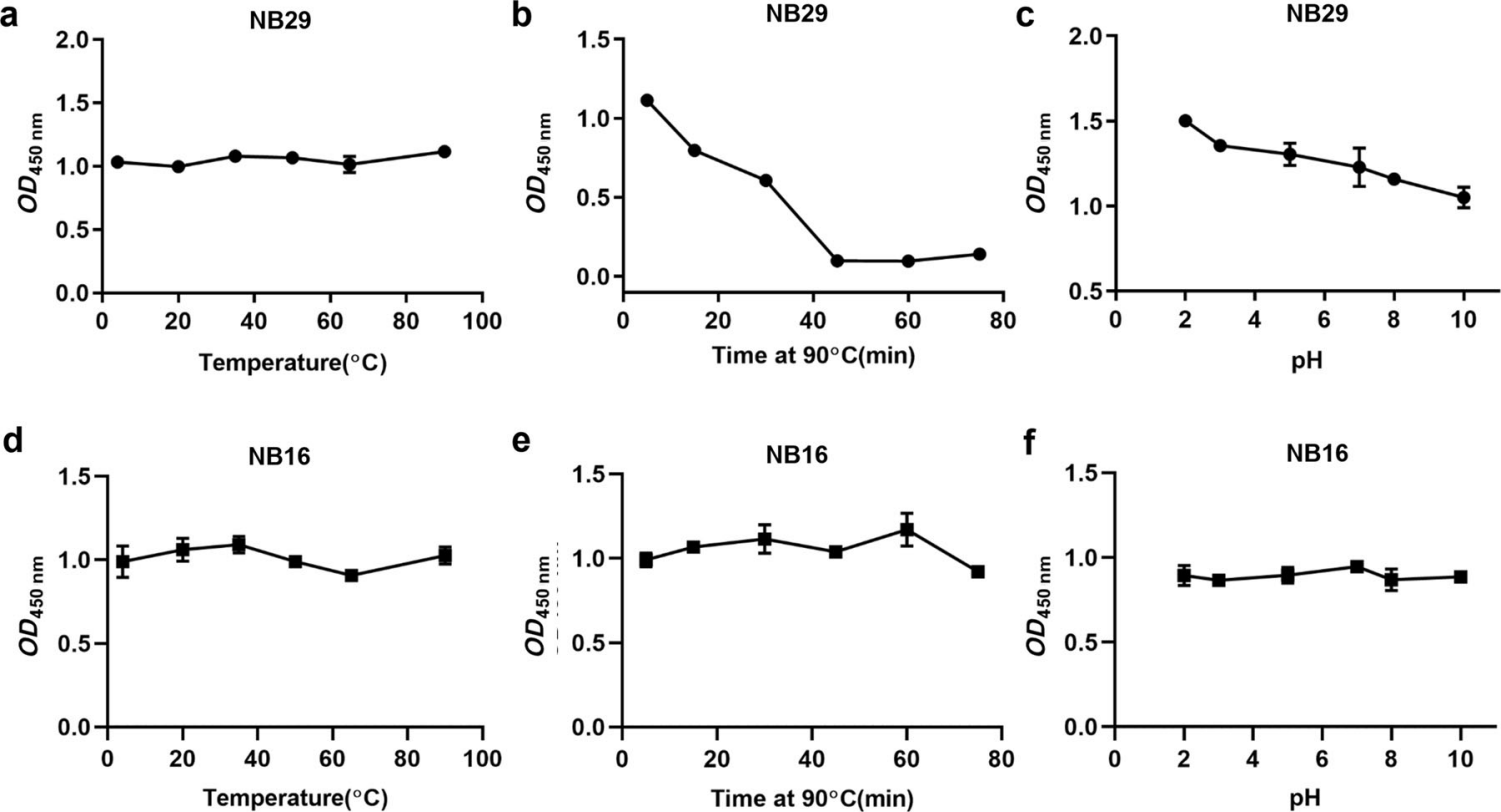
Analysis of thermal and chemical stability for NB29 and NB16. **a**, **d** NB29 and NB16 were incubated for 5 min at different temperatures (4, 20, 35, 50, 65, and 95 °C), and the binding capacities to FGL1 was detected by ELISA. **b**, **e** NB29 and NB16 were incubated at 90 °C for 5 min, and the binding capacities to FGL1 were detected by ELISA. **c**, **f** NB29 and NB16 were incubated in different pH buffers for 2 h, and the binding capacities to FGL1 were detected by ELISA.

NB29 and NB16 have remarkable stabilities under different temperature and pH conditions. As shown in Fig. 5, the binding activities of NB29 and NB16 remained stable after incubation at 90 °C for 5 min (Fig. 5a and d). Moreover, NB29 binding capacity remained at 50% after incubation at 90 °C for 30 min (Fig. 5b), and the binding activity of NB16 was not substantially affected by long-term treatment at 90 °C (Fig. 5e). NB29 also showed considerable stability in acidic environments, but its resistance to basic pH is not strong. The binding activity of NB29 was reduced to 71% after 2 h of incubation at pH 10 (Fig. 5c). However, the change in pH did not reduce the binding activity of NB16 to FGL1 (Fig. 5f).

### Preparation of HRP-labeled nanobody conjugate

**Fig. 6:**
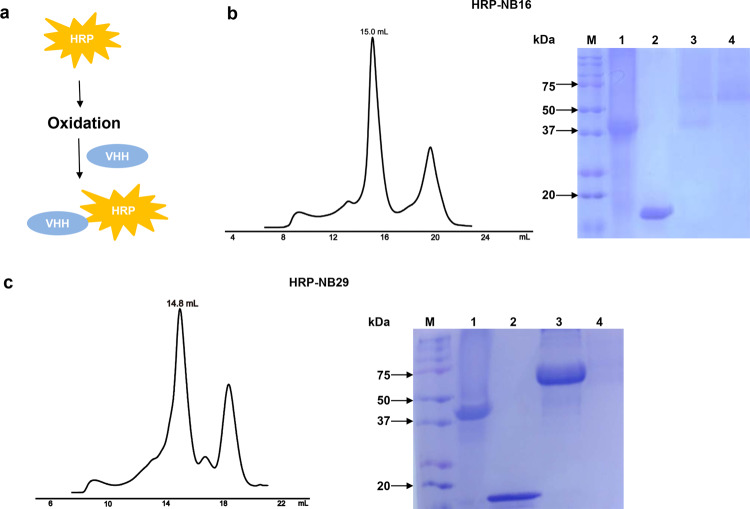
Conjugation of HRP to nanobodies. **a** Schematic illustration of strategies to conjugate HRP to nanobodies. **b** Size-exclusion chromatography profile of the HRP-NB16 conjugation mixture. HRP-NB16 conjugation was analyzed by SDS-PAGE. Lane 1, HRP; lane 2, NB16; lane 3, HRP-NB16 conjugation mixture through SEC; lane 4, purified HRP-NB16 conjugate. **c** Size-exclusion chromatography profile of the HRP-NB29 conjugation mixture. HRP-NB29 conjugation was analyzed by SDS-PAGE. Lane 1, HRP; lane 2, NB29; lane 3, HRP-NB29 conjugation mixture through SEC; lane 4, purified HRP-NB29 conjugate.

HRP was conjugated with nanobodies by periodate oxidation, as recently reported [33]. A schematic illustration of the strategy to label nanobodies with HRP is displayed in Fig. 6a. As shown in Fig. 6b, the conjugates and unconjugated nanobodies were clearly separated through SEC, but SDS-PAGE indicated that HRP was labeled with NB16 at different ratios. Free HRP was still present as the main peak. After purification on a Ni-NTA column, SDS-PAGE showed a band of ~60 kDa corresponding to the conjugates (HRP: nanobody=1:1), and there was no band of 44 kDa. The purified sample was selected as the detector since fewer nanobodies per HRP would increase the signal to noise ratio. The result of HRP conjugation with NB29 was similar to that for NB16 (Fig. 6c).

### Analysis of the assay performance with spiked samples

**Fig. 7:**
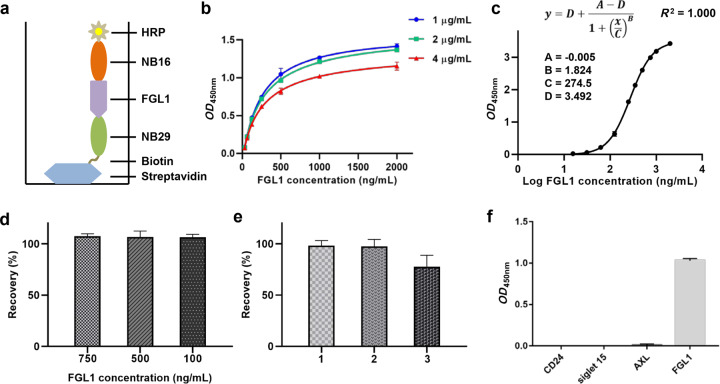
Detecting FGL1 by nanobody-based immunoassay and study on the recoveries of FGL1. **a** Schematic diagram of dual nanobody detection of FGL1. Biotin-NB29 as a capture and HRP-NB16 as a probe. **b** Comparison of varying concentrations (1, 2, and 4 µg/mL) of biotin-NB29. Other invariable conditions: streptavidin, 2 μg/mL coated overnight at 4 °C; HRP-NB16, 1:50 dilution. **c** A four-parameter logistic model for calibration curve fitting was used to analyze the FGL1 concentration over a range of 15.625 ng/mL to 2000 ng/mL. **d** Recoveries of FGL1 from different concentrations (750, 500, and 100 ng/mL). **e** Recoveries of the spiked sample in three different human sera. **f** Specificity tests with several unrelated biomarkers (CD24, siglet 15, and AXL).

As illustrated in Fig. 7a, we successfully constructed a streptavidin-bridged double-nanobody ELISA using the following steps. Streptavidin was coated directly on the plate, biotin-NB29 was added as the capture nanobody, and different concentrations of FGL1 were added. Finally, the HRP-NB16 conjugate was added as a detector. To obtain sufficient signal and low background, the working concentrations of biotin-NB29 and HRP-NB16 were optimized for the assay. As shown in Fig. 7b and c, we tested different concentrations of biotin-NB29 (4, 2, 1 μg/mL) and HRP-NB16 (1:20, 1:50, 1:100) for sandwich ELISA. Eventually, the optimal working concentration of biotin-NB29 as the capture nanobody was found to be 1 μg/mL, and that for the HRP-NB16 conjugate as the detector was 1:50. Thus, the calibration curve was analyzed according to a four-parameter logistic model over a range of FGL1 concentrations from 15.625 to 2000 ng/mL (Fig. 7d), and the bias of the back-calculated concentration of the calibration curve was within the range of ±20%. It demonstrated a high sensitivity with working concentrations of streptavidin, biotin-NB29 and HRP-NB16 of 2, 1 μg/mL and 1:50 dilution, respectively.

To evaluate the accuracy and precision of the assay by analyzing spiked samples, quality control samples (750, 500, and 100 ng/mL) were tested, and the recovery values were 107.4%, 106.6%, and 106.3%, respectively (Fig. 7e), indicating the good accuracy of the assay. We also investigated the recovery of FGL1 in human serum samples by spiking 500 ng/mL FGL1 into three individual human serum samples. The recovery of these samples was 98.2%, 97.5%, and 77.9%, respectively, for spiked samples prepared in a 1:10 dilution of the sample matrix (Fig. 7f). Furthermore, specificity evaluation was performed to investigate the possible cross-reactivity of selected nanobodies with other unrelated biomarkers (CD24, siglet 15, and AXL). The results showed that the three unrelated biomarkers (100 ng/mL) yielded signal below the lower limit of quantification of the assay (15.625 ng/mL), while 100 ng/mL FGL1 could be detected accurately (Fig. 7g). Our results indicated the reliability and accuracy of the double nanobody ELISA method for the detection of FGL1 in human serum.

## Discussion

Immunotherapies represent a significant breakthrough in the cancer treatment paradigm. Blocking the PD-1/PD-L1 immune checkpoint has shown promising efficacy in various malignant tumors [34–36]. FGL1/LAG-3 may be another promising immune checkpoint. A recent study demonstrated that agents targeting the FGL1/LAG-3 pathway could stimulate tumor immunity and inhibit tumor growth [12]. FGL1 and LAG-3 are closely related to the prognosis and clinicopathological features of various types of cancer [37], and clinical studies have shown that dual FGL1/LAG-3 and PD-1/PD-L1 blockade therapy has promising survival benefits and long-duration response rates [38]. However, not all patients responded to the treatment, and the reactivity was related to their expression level prior to treatment. To date, there is no noninvasive tool available to accurately evaluate the relevant expression in vivo. Recently, Nick Devoogdt et al. developed a method to evaluate mouse LAG-3 expression using single-photon emission computed tomography (SPECT)/CT imaging [11]. The method was required to inject ^99m^technetium-labeled nanobodies against LAG-3 for diagnosis, which may pose risks of complications. While FGL1 is secreted into the circulation system and its plasma level is thus logically correlated with the advancement of the disease, it is an ideal biomarker for assay development to analyze its expression to diagnose and monitor the therapeutic outcome. Here, we developed a double nanobody sandwich ELISA for human FGL1 detection as a cancer diagnostic.

This is the first report of human FGL1 expression and purification. We have tried many methods of expressing FGL1 in different systems, but the expression level is very low. It was found that the expression of FGL1 increased when fused with the human IgG1 Fc fragment. The fusion of IgG1 Fc with the target protein can be used as a general method for the expression and purification of many proteins and is helpful for increasing the protein yield, facilitating the purification and maintaining the functions of these proteins. In addition, the size exclusion chromatography profile showed that the secreted FGL1-Fc folded well and appeared in multiple states. In future research, we will need to test which state is the physiological active state and interacts with LAG-3.

We successfully screened several nanobodies targeting FGL1. The generation of Nbs through phage display technology is faster than that of conventional polyclonal and monoclonal Abs. In addition, these nanobodies show some striking advantages over conventional antibodies, such as high affinity and high stability and solubility. Most of the nanobodies we screened had affinity in the nanomolar range and were still active even after treatment at 90 °C for 5 min. All of these excellent features are conducive to the development of biosensors.

However, the plasma FGL1 level is very low, and a reliable quantitative method with high sensitivity and specificity is required for its detection. Double nanobody-based sandwich ELISA requires a pair of nanobodies targeting different epitopes of FGL1 with high affinity and specificity, one as a capture and the other as a probe. The selected NB29 nanobody with the strongest binding to FGL1 was biotinylated and directionally captured by streptavidin coated on a MaxiSorp plate, and the biotinylation of the capture nanobody improved the sensitivity. The sensitivity of directional ELISA is higher than that of conventional undirectional ELISA by passive adsorption of the nanobody. As reported, a polyHRP-labeled nanobody conjugate as a detection system can be favorably prepared through an improved periodate oxidation reaction under low temperature [33]. As expected, polyHRP coupled with another nanobody (NB16) as the reporter in this much milder environment and showed higher sensitivity. The sensitivity of the current method can be further explored via more sensitive platforms, such as electrochemiluminescence (ECL) and homogeneous time-resolved fluorescence (HTRF) platforms.

In summary, we developed a well-optimized double-nanobody sandwich ELISA for the detection of FGL1 and the primary exploration of the factors contributing to the performance of quantification detection.
